# Repulsive Aftereffects of Visual Space

**DOI:** 10.3390/vision7040073

**Published:** 2023-11-15

**Authors:** Eckart Zimmermann

**Affiliations:** Institute for Experimental Psychology, Heinrich Heine University Düsseldorf, Universitätsstraße 1, 40225 Düsseldorf, Germany; eckart.zimmermann@hhu.de

**Keywords:** visual adaptation, aftereffects, space perception

## Abstract

Prolonged exposure to a sensory stimulus induces perceptual adaptation aftereffects. Traditionally, aftereffects are known to change the appearance of stimulus features, like contrast, color, or shape. However, shifts in the spatial position of objects have also been observed to follow adaptation. Here, I demonstrate that visual adaptation produced by different adapter stimuli generates a bi-directional spatial repulsion. Observers had to judge the distance between a probe dot pair presented in the adapted region and compare them to a reference dot pair presented in a region not affected by adaptation. If the probe dot pair was present inside the adapted area, observers underestimated the distance. If, however, the dot pair straddled the adapted area, the distance was perceived as larger with a stronger distance expansion than compression. Bi-directional spatial repulsion was found with a similar magnitude for size and density adapters. Localization estimates with mouse pointing revealed that adaptation also affected absolute position judgments. Bi-directional spatial repulsion is most likely produced by the lines of adapter stimuli since single bars used as adapters were sufficient to induce spatial repulsion. Spatial repulsion was stronger for stimuli presented in the periphery. This finding explains why distance expansion is stronger than distance compression.

## 1. Introduction

All primary visual features are adaptable (for a review, see [1]). The prolonged exposure of a certain adapter stimulus produces an aftereffect such that a feature of a subsequently presented test stimulus is changed in appearance. This aftereffect might be positive in the direction of the adapter’s appearance or negative, repelling the appearance of the test stimulus away from that of the adapter stimulus. The classical example is the waterfall illusion [2], and a more recently discovered instance is adaptation to body orientation [3]. Classical models [4,5,6] suggest neuronal fatigue, i.e., the elevation of the neuron’s threshold for the activation of potential action [7] as the basis of adaptation. To explain negative aftereffects, fatigue models assume that adaptation reduces the responses of those channels most sensitive to the adapter stimulus in a population of narrowly tuned, overlapping channels [6].

Another influential theory tries to explain adaptation as efficient neuronal coding in response to redundant sensory information. Gibson [8] already suggested that the repulsive tilt aftereffect after adaptation to oblique lines might be considered a recalibration of spatial orientation. This idea has been developed further, granting visual adaptation a functional role. During adaptation, neuronal responses might undergo self-calibration and decorrelation in order to promote the effective use of its narrow range [9]. Shifting neuronal responses after prolonged stimulus exposure could improve discrimination and also increase the salience of novel stimuli. Images in the natural world are highly structured and redundant [10]. Tuning neuronal responses to image statistics would increase coding efficiency [11]. This theory works well for visual features because adaptive repulsion exaggerates their differences and thereby enhances discriminability. However, it is unclear what this theory could predict for the encoding of space since spatial positions have to be represented veridically for most purposes, such as guiding movements. 

Studies on figural aftereffects were the first to investigate the effect of prolonged stimulus exposure on size and position [12,13]. Presenting an object with a given size provokes a repulsive aftereffect, such that the size of subsequently flashed probe objects is pushed away from the adapter size [14,15]. An fMRI investigation into this behavioral effect revealed V1 as the most likely candidate to host the corresponding neural adaptation [15]. A more recent study found that size adaptation was unaffected by perceived size and binocular disparity, suggesting that it occurs as early as the lateral geniculate nucleus [16]. After texture density adaptation, the distance between two probe dots appeared to be shrunk [17]. 

The available theories on adaptive visual repulsion suggest that neural encoding of size or shape influences how we judge the relative distance of successively presented objects. However, adaptation to visual stimuli could directly distort an internal spatial metric before the object size or shape is processed. Since this distortion takes place at an early level of the neural hierarchy [15,16], it could be inherited in position judgments or estimations of size or shape. In this study, I sought evidence for a distortion of spatial metrics via visual adaptation. In this view, the borders of the adapter stimuli, as the region with the highest contrast, might locally fatigue activity in a neuronal population representing a labeled-line code of space. As a consequence, spatial encoding could be inhibited at the adapted, i.e., fatigued, neurons, thus giving higher weight to the response of neighboring neurons. In this way, adaptive fatigue could result in a transient misrepresentation of space around the region of the borders of the adapter stimuli. I aimed to test this theory against existing accounts of adaptive distortions of visual space. First, I tested localization for stimuli that either straddled the adapted region or were placed inside the adapted region. By definition, repulsion implied a compression for the former and an extension for the latter stimuli. Second, I tested how a single bar stimulus—that should mimic only the border of the size adapter—affected spatial localization. Third, by testing localization with a mouse pointer rather than a comparison task, I tested whether adaptation influenced the absolute apparent position. 

## 2. Methods Section

### 2.1. Participants

In Experiment 1, 6 subjects (4 female, 2 male, mean age = 32 years); in Experiment 2, 4 subjects (3 female, 1 male, mean age = 29 years) and in Experiment 3, 6 different subjects (3 female, 3 male, mean age = 30 years) participated. All subjects were reported to have normal or corrected-to-normal vision. Experimental procedures were approved by the local ethics committee of the psychological department of the Heinrich-Heine University Düsseldorf. Written informed consent was obtained prior to each experiment in accordance with the declaration of Helsinki.

### 2.2. Apparatus

Subjects were seated 45 cm from an Eizo FlexScan T57S with the head stabilized via a forehead rest. The visible screen diagonal was 20 in., resulting in a visual field of 40 dva × 30 dva. Stimuli were presented on the monitor with a vertical frequency of 120 Hz, at a resolution of 800 × 600 pixels, on a homogeneous gray background. 

### 2.3. Trial Structure of Experiments

Trials in baseline sessions started with the presentation of a probe and a standard stimulus. Both stimuli were presented simultaneously for 100 ms. Trials in adaptation sessions started with the presentation of an adapter stimulus for 5000 ms and were always present on the right side of the screen (stimulus center: x: 10 dva, y: 0 dva). The stimulus center was defined as x: 0 dva and y: 0 dva. After the offset of the adapter, only the fixation point was visible for 100 ms. Then, the probe and the reference stimuli were shown simultaneously for 100 Ms.

#### 2.3.1. Experiment 1

Experiment 1 investigated the influence of size or density adaptation on the perceived spatial distance of two simultaneously flashing dots. In baseline sessions, a fixation point (0.5 dva × 0.5 dva) was presented continuously throughout all experimental sessions in the screen’s center, and participants were required to keep their gaze directed at it. After 1000 ms, a probe stimulus was shown on the right side of the screen (stimulus center: x: 10 dva, y: 0 dva), and simultaneously, a standard stimulus was shown on the left side (stimulus center: x: −10 dva, y: 0 dva). The probe and reference stimuli each consisted of two dots (radius: 0.25 dva). Both stimuli were shown for 100 ms. The orientation of the probe and the reference dot pairs was identical on a given trial but randomly selected across trials out of 4 possible orientations (0–180 dva in steps of 45 dva). The change in orientation for the dot pairs served to avoid the repetition of the same orientation across the trials, and it was not analyzed separately. In all trials, the distance between the reference dots was either 4 dva or 12 dva, and the distance between the probe dots was randomly selected across the trials (−1.5 dva—1.5 dva + reference dot distance, in 7 equidistant steps, each presented 10 times). After the disappearance of the probe dot pair, participants were required to report if the probe pair distance was bigger on the left or right side by pressing the corresponding arrow keys on the computer keyboard. Then, the next trial started. 

**Figure 1:**
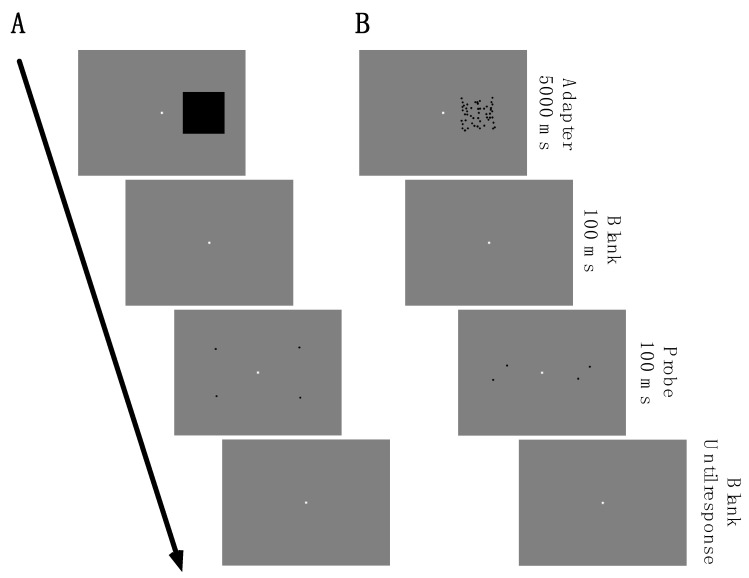
(**A**) Graphical illustration of the trials’ structure in Experiment 1. A trial started with the presentation of a size adapter (8 dva × 8 dva) for 5000 ms. After a blank of 100 ms, the probe and reference stimuli were shown simultaneously for 100 ms, consisting of a dot pair. The probe had a constant inter-dot distance of either 4 dva or 12 dva. (**B**) The procedure to measure distance judgments after density adaptation. The effects of size and density adaptation were measured in separate sessions. The density adapter consisted of 50 dots that were arranged relative to an invisible grid. Each dot was displaced randomly by 0.3 dva every 300 ms. Except for the features of the adapter stimulus, the procedure was identical as in (**A**).

Adaptation sessions were measured after the baseline sessions. In adaptation sessions, trials started with the presentation of either a size (Figure 1A) or a density (Figure 1B) adapter. The size adapter consisted of a rectangle (8 dva × 8 dva) and changed in luminance from white to black every 300 ms to avoid the creation of an afterimage. The density adapter consisted of 50 dots (radius: 5 pixels) that were randomly positioned in an area of 8 dva × 8 dva with a minimum distance of 0.75 dva between each other. Every 300 ms, each dot was displaced by up to 0.3 dva in a random direction. The adapters were shown for 5000 ms. After the adapter offset, the screen remained blank for 100 ms. Then, the probe dot pair was presented on the right side simultaneously with the reference dot pair that was shown on the left side. When the subject gave the response, the next trial started. In Experiment 1, each subject completed 420 trials (70 for each psychometric function × 3 session types (baseline, 4 dva, 12 dva dot distance) × 2 conditions (numerosity/density)). Each psychometric function was measured in a separate experimental block. 

#### 2.3.2. Experiment 2

**Figure 2:**
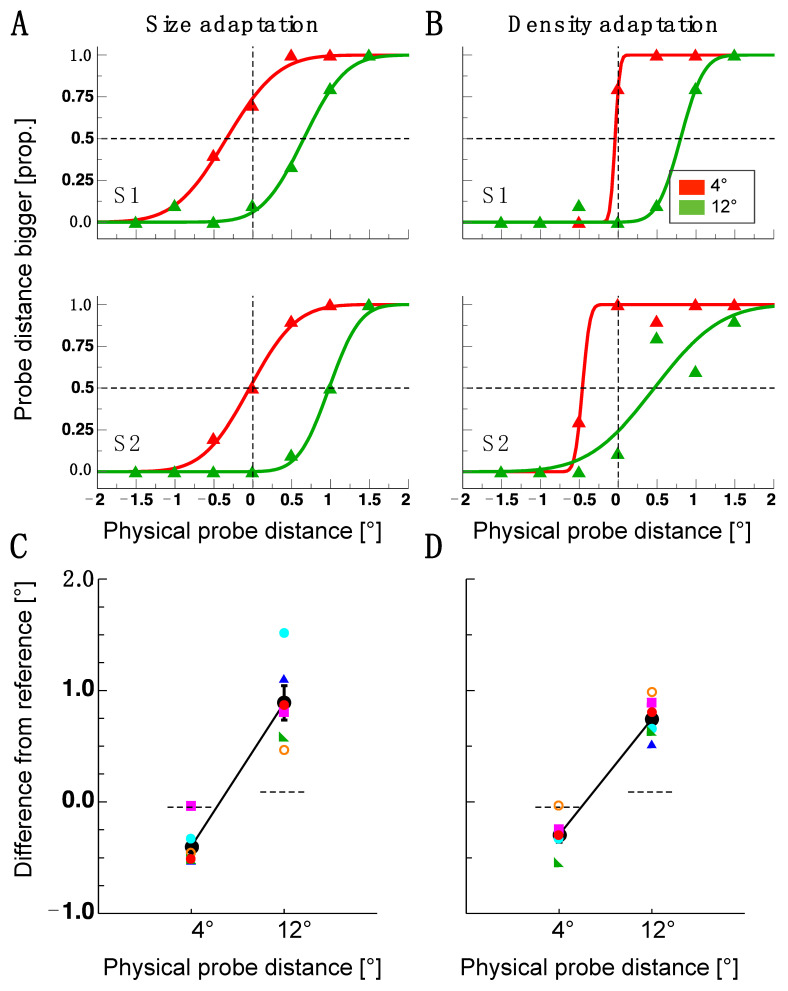
Results of Experiment 1. (**A**) Example psychometric functions from the size adaptation experiment. Data in red show the results from a 4 dva dot pair distance, and data in green results from a 12 dva dot pair distance. (**B**) Example of psychometric functions from the density adaptation experiment. Same conventions as in 12 dva. (**C**) Single subject and average distance judgments for the probe dot pairs (4 dva and 12 dva) after size adaptation. Colored symbols show single-subject results and black disks show average results. Error bars are the standard error of the sample mean. The dashed line shows a comparison between the probe and reference pair measured at the baseline. (**D**) Single subject and average distance judgments for the probe dot pair after density adaptation. Same conventions as in (**C**).

Experiment 2 tested whether the presentation of the size adapter border could suffice to induce spatial mislocalization. To this end, adapter stimuli consisted of a single bar (width: 10 dva, height: 0.25 dva), presented either at the position of the upper (x: 10 dva, y: 4 dva) or the lower (x: 10 dva, y: −4 dva) border of the adapter stimulus that was used in Experiment 1. Probe stimuli were identical to those of Experiment 1 with probe pair distances of 4 dva and 12 dva. Except for the change in the adapter stimulus, the procedure of Experiment 2 was identical to that in Experiment 1. Figure 2 shows the configuration of small and large probe dot pair distances and the positions of the adapter stimuli. Four adaptation conditions were tested in separate sessions as follows: (i) only the lower bar, (ii) only the upper bar, (iii) both bars simultaneously, and (iv) both bars simultaneously with a higher vertical eccentricity ((x: 10 dva, y: −8 dva) and (x: 10 dva, y: 8 dva)). In Experiment 2, each subject completed 700 trials (70 for each psychometric function × 4 session types × 2 conditions (numerosity/density) + 2 × 70 baseline trials). Each psychometric function was measured in a separate experimental block.

#### 2.3.3. Experiment 3

In Experiment 3, adapters were density stimuli consisting of random dots distributed equally over a rectangular area (8 dva × 8 dva). Probe stimuli consisted of one single dot per trial that had to be localized with a mouse pointer. As this experiment tested absolute localization, no reference stimulus was shown. The probe dot appeared in one of 8 possible locations (x: 2 dva, y: 0 dva), (x: 1 dva, y: 0 dva), (x: −1 dva, y: 0 dva), (x: −2 dva, y: 0 dva),(x: 0 dva, y: 2 dva), (x: 0 dva, y: 1 dva), (x: 0 dva, y: −1 dva), (x: 0 dva, y: −2 dva). Probe locations were tested pseudo-randomly across the trials, and each was repeated 10 times. In Experiment 2, each subject completed 160 trials (10 for each location × 8 locations × 2 conditions (before/after adaptation)).

## 3. Data Analysis

The responses for each experimental increment were averaged within each participant. Each psychometric function was based on 70 trials (10 repetitions for each stimulus level). Cumulative Gaussian functions were fitted to these psychometric data. The point of subjective equality was chosen as the increment level at which the performance reached 50%. The mean and SEM were estimated across subjects. 

## 4. Results

Participants judged the spatial distance between two probe dots after being exposed to an adapter stimulus for 5000 ms. Distance estimates were indicated in comparison to a reference stimulus that was presented in an opposite visual field that was not adapted. In Experiment 1, a size adapter and a density adapter were used in separate sessions. The adapters had a size of 8 × 8 dva. Figure 2A,B show psychometric functions for two example observers. Data in red represent the results from trials in which the distance between the probe dots was 4 dva, and data in green represent results from trials in which it was 12 dva. The abscissae show the difference in the distance of the physical probe dot pair compared to the reference dot pair on the side that was not adapted. Negative numbers indicate underestimations of the probe dot pair distance, and positive numbers indicate overestimation. After size and density adaptation, both subjects shown in Figure 2A,B judged the distance of the 4 dva probe dot pair to be smaller than the reference dot pair. The 4 dva probe dot pair lies perfectly within the area of the adapter stimuli, whereas the 12 dva probe dot pair straddles the adapted area. Both participants judged the distance of these latter stimuli to be larger than the reference stimuli. For observer S1, the size adaptation effect produced a larger shift in the bias from the veridical (0 dva) of the 12 dva probe dot pair than for the 4 dva probe dot pair. For observer S2, the 4 dva probe dot pair was not affected by size adaptation, whereas the 12 dva probe dot pair shifted from veridical. Density adaptation had almost no effect for the 4 dva probe dot pair and a similar effect as size adaptation was observed for the 12 dva probe dot pair in observer 1. In observer 2, both the 4 dva and the 12 dva probe dot pair were affected by density adaptation. Figure 2C,D show points of subjective equivalence (PSEs) for all observers and the resulting average judgments. For size adaptation, all observers underestimated the 4 dva dot distance and overestimated the 12 dva dot distance. These biases had nearly the same magnitude. Similarly, after density adaptation, all observers underestimated the 4 dva dot distance, and all observers overestimated the 12 dva dot distance. 

To estimate statistically whether visual adaptation evoked a bi-directional spatial shift, I tested the results from the 4 dva and the 12 dva dot distances against their respective baselines with paired *t*-tests (size-adaptation 4 dva: *t*(5) = −4.55, *p* = 0.006; 12 dva: *t*(5) = 5.18, *p* = 0.004; density adaptation: 4 dva: *t*(5) = −3.73, *p* = 0.014; 12 dva: *t*(5) = 9.20, *p* = 0.0003). The effect sizes were d = 0.92 for size adaptation and d = 1.09 for density adaptation. The results from Experiment 1 show that adaptation influenced spatial distance judgments. However, since these effects were similarly strong for size and density adaptation, they did not determine if a particular feature of the respective adapters was responsible for the effect. As the density adapter also had a definite size, judgments might be biased only by adaptation to size. 

**Figure 3:**
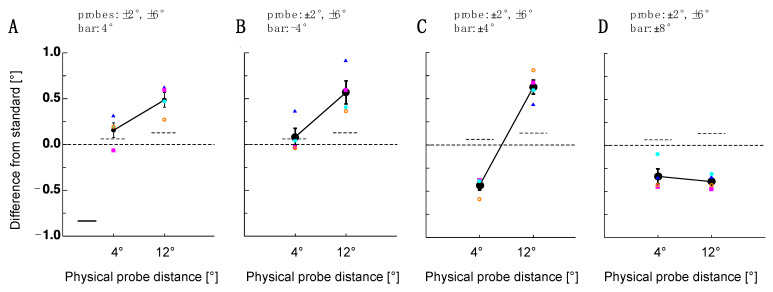
Results of Experiment 2. (**A**) Single subject (colored symbols) and average distance judgments (shown in black) for the probe dot pair after adaptation to the upper bar. Error bars are the standard error of the sample mean. The dashed line shows a comparison between the probe and reference pair measured at the baseline. (**B**) Single subject (colored symbols) and average distance judgments (shown in black) for the probe dot pair after adaptation to the lower bar. The same conventions as in 3A. (**C**) Single subject (colored symbols) and average distance judgments (shown in black) for the probe dot pair after adaptation to the lower and the upper bar are presented simultaneously. The same conventions as in 3A. (**D**) Single subject (colored symbols) and average distance judgments (shown in black) for the probe dot pair after adaptation to the lower and the upper bar presented simultaneously at a higher eccentricity. The same conventions as in (**A**).

Adaptation-induced object repulsion is bi-directional. The sign of the repulsion direction depends on the position of the probe object relative to the adapter outline. In this interpretation, spatial compression inside the adapted area was the consequence of a dot pair that was flanked by adapter stimulus borders. However, in conditions where the distance between the object pair extended, the adapter stimulus outline fell between the two dots. To test the hypothesis that only the adapter outline was relevant for object repulsion to occur, in Experiment 2, a single bar was presented. This bar was shown at either the upper or lower vertical location of the adapters used in Experiment 1 (see Figure 2). For both adapter locations, a 4 dva and a 12 dva dot pair distance was tested. As can be seen in Figure 3A,B, for the upper and lower bar, object repulsion could only be observed for the 12 dva dot pair distance but not for the 4 dva dot pair distance. The statistical significance of post-adaptation spatial estimates was tested using paired *t*-tests against the baseline performance (lower bar 4 dva: *t*(3) = 1.19, *p* = 0.32; 12 dva: *t*(3) = 4.44, *p* = 0.02; upper bar: 4 dva: *t*(3) = 0.23, *p* = 0.83 12 dva: *t*(3) = 3.49, *p* = 0.04).

If object repulsion via lines works only for objects presented in the periphery (dot pair—distance: 12 dva), it does not explain why size or density adapters also shrink from the distance of dot pairs presented in the foveal region. To examine this issue more closely, in Experiment 2, I presented the upper and lower bar simultaneously in order to mimic the horizontal lines of the size or density adapters (see Figure 3C). Indeed, with this adapter stimulus, I found significant shrinkage for the small dot pair distances (*t*(3) = −9.65, *p* = 0.002) and the significant expansion of large dot pair distances (*t*(3) = 6.49, *p* = 0.007). 

Why are dot pair distances more susceptible to adaptation if they are placed outside the adapter region? The following two factors might play a role: the absolute eccentricity of the probe stimuli or the distance between the adapter and probe stimulus. The first factor, i.e., the absolute eccentricity, is likely relevant because many adaptation effects are stronger in the periphery [18,19,20,21,22,23,24,25,26]. The second factor, i.e., the distance between the adapter and probe stimulus, has been suggested by [12]. To dissociate both factors, I presented the upper and lower bar simultaneously with a relative distance of 14 dva. Under this condition, both probe dot pairs, the small and the large distance, were flanked by the adapter stimuli. However, the distance to the adapter stimulus was higher for the small than for the large probe dot pairs. With this stimulus setup, I found significant spatial shifts for both the small (*t*(3) = −4.86, *p* = 0.016) and the large (*t*(3) = −13.58, *p* < 0.001) probe dot pair distances (see Figure 3D). 

**Figure 4:**
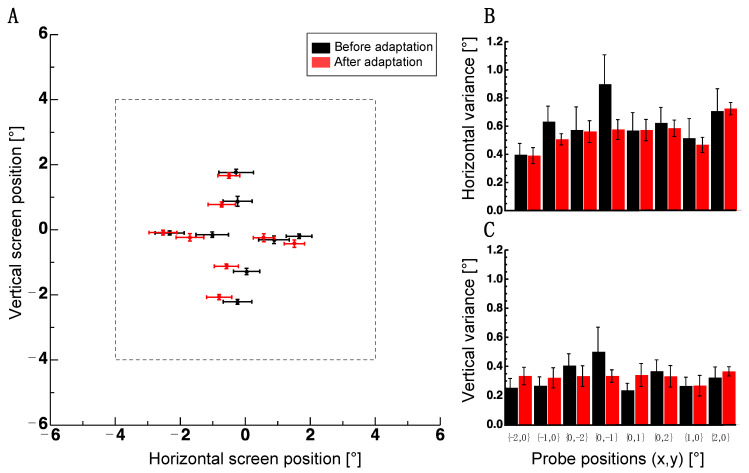
Results of Experiment 3. (**A**) The average localization of probe dots before (black disks) and after adaptation (red disks). Error bars are the standard error of the sample mean. The dashed line indicates the borders of the adapter. (**B**) Average standard deviations for horizontal localizations of probe positions. The same conventions as in 4A. (**C**) Average standard deviations for vertical localizations of probe positions. The same conventions as in (**A**).

For the probe stimuli used so far, subjects had to compare the relative distances between dots. In Experiment 3, I aimed to assess whether adaptation changed absolute spatial positions. To this end, subjects were asked to indicate the perceived positions of probe dots with a mouse pointer. Figure 4A shows average localization in Experiment 3 before (shown in black) and after adaptation (shown in red). An adaptive shift can be seen for dots spread along the vertical meridian (x,y coordinates: 0, −2; 0, −1; 0, 1; 0, 2). After adaptation, the dots in the upper and the lower visual fields were perceived to be shifted more inward compared to dots measured before the adaptation. This pattern is consistent with a repulsion of dot positions from the adapter border. By contrast, dots spread along the horizontal meridian (x,y coordinates: −2, 0; −1, 0; 1, 0; 2, 0) did not appear to shift repulsively. For the vertical localization component, a non-parametric repeated measures ANOVA with the factors adaptation (before/after) and probe positions (eight positions) confirmed a significant interaction between the factors of adaptation and probe positions (F(7,35) = 2.452, *p* = 0.037). No significant main effect was found (adaptation: F(1,5) = 2.519, *p* = 0.703; probe positions: F(7,35) = 1.259, *p* = 0.298). No significant effect was found in the horizontal localization component (adaptation: F(1,5) = 2.52, *p* = 0.173, probe positions: F(7,35) = 1.189, *p* = 0.333, interaction: F(7,35) = 1.987, *p* = 0.085).

A reason for the absence of an effect in the horizontal component might be the higher variance in that orientation (see Figure 4B,C). A non-parametric repeated measures ANOVA on the variances with factor orientation (horizontal/vertical), adaptation (before/after) and probe positions (8 positions) revealed the significant main effect of the factor orientation (F(1,5) = 35.938, *p* = 0.002), adaptation (F(1,5) = 10.633, *p* = 0.022) and a significant two-way interaction effect (F(1,5) = 20.918, *p* = 0.006) between orientation and adaptation. The factor positions (F(7,35) = 1.274, *p* = 0.292) and the remaining interactions (positions/adaptation: F(7,35) = 0.976, *p* = 0.464) positions/orientation: F(7,35) = 0.558, *p* = 0.785 positions/adaptation/orientation: F(7,35) = 1.487, *p* = 0.204) were not significant.

## 5. Discussion

In this study, I found that both size and density adaptation induced a bi-directional spatial repulsion. Two objects were either placed inside the adapted area or straddled the adapted area. When presented inside the adapted area, objects were perceived to be closer to each other. When straddling the adapted area, the distance between the objects expanded. Spatial expansion aftereffects were stronger than spatial compression. These effects can be explained by the repulsion of objects from the border of the adapter stimuli. To test this hypothesis, I presented a single bar as an adapter. This stimulus produced adaptive spatial shifts for large but not for small object distances. With two bar adapters that mimicked both the vertical border of the size or density adapters, repulsion was observed for both distances. Two factors might be responsible for this difference as follows: first, the eccentricity of the probes and, therefore, the activation of more peripheral retinal regions, and second, the distance of the probes to the adapter stimuli. The first hypothesis is likely because visual adaptation is known to generate stronger aftereffects in the periphery, including tilt aftereffects [18,19], motion aftereffects [20,21], shape aftereffects [22,23], face aftereffects [24,25] and contrast aftereffects [26]. Under the second hypothesis, adaptation might be absent (as it was in Experiment 2) because stimuli are presented closer to the adapter, as has already been proposed by [12]. This idea could be directly tested by repeating the experiment with adapters placed farther into the periphery. With this manipulation, the small and the large probe distances were flanked by the adapters. Although these two probe pairs had different distances to the adapter stimuli, the spatial repulsion magnitude was almost identical for both probe pairs. Thus, data demonstrate that visual adaptation produces spatial repulsion across the visual field that grows in the periphery. A further experiment, in which subjects localized probes with the mouse cursor, confirmed that adaptation did not only affect relative object distances but absolute spatial localization. 

Changes in apparent size and position following the presentation of smaller or larger objects were first demonstrated in classical studies on figural aftereffects [12,13]. Ganz [27] later argued that repulsive aftereffects resulted from the presence of an afterimage. My experiments rule out this hypothesis. The adapter stimulus in the current study changed luminance every 300 ms to avoid the induction of an afterimage. Yet, repulsion was still observed. Changes in perceived density have been suggested recently as cues for spatial shifts based on the finding that adaptation to a visual texture induces compression for the apparent distance of two dots [17]. In the current study, I replicated these findings and showed that, as for size adaptation, object distances compress when presented inside the adapted area and expand when straddling the adapted area. Explaining this bi-directional spatial aftereffect with changes in perceived density requires the apparent density to decrease inside the adapted area and increase adjacent to it. Otherwise, density adaptation cannot explain the expansion of object distances. Thus, changes in density perception cannot account for the bi-directionality of visual shifts. It has been reported recently that density adaptation can induce bi-directional shifts when probe and reference stimuli are presented sequentially and not simultaneously [28]. Density adapters, like most other visual feature adapters, produce negative aftereffects. In order to obtain an increase in perceived density in the probe stimulus, the adapter stimulus has to be presented with a lower density than the probe stimulus. However, in my study, the very same adapter density induced a bi-directional spatial shift. The direction of effects between density adaptation and spatial perception was, thus, dissociated by this experiment. 

Adaptation to the border of a size adapter can easily explain bi-directionality. It is known that adaptation to size is followed by a bi-directional repulsion of the test stimuli. Objects that are smaller than the adapter appear compressed, and objects larger than the adapter appear to be expanded. A population of neurons encoding size might alter our ability to judge spatial distances between objects. However, this interpretation is hindered by the finding that absolute spatial positions are also affected by visual adaptation. Experiment 2 of the current study showed that a single bar, which mimicked the borders of size adapters, was sufficient to induce object repulsion. The easiest explanation for spatial repulsion is, therefore, an adaptive distortion in a labeled line code of visual space. Bi-directional spatial repulsion is consistent with fatigue models of adaptation. These models suggest that adaptation reduces the firing rate of those neurons in a population of narrowly tuned feature-selective channels that are most sensitive to the features of the adapter stimulus [6]. This selective reduction explains negative aftereffects in which the probe object’s appearance is repelled from that of the adapter stimulus. The same rationale can be applied to a population of neurons representing visual space. Adaptation to a border or a bar might transitorily depress neuronal firing at the adapted location. This depression can lead to a shrinkage in space at the adapted position and a stretching of space close to the adapted location. 

Spatial repulsion following adaptation has also been found in other sensory domains as follows: adaptation to an auditory stimulus presented with a time delay at one of the ears leads probe sounds to be mislocalized in the opposite direction than the adapter [29]. Prolonged exposure to vibro-tactile stimulation led to an overestimation of the separation of tactile impulses that straddled the adapted region [30].

A limitation of the current study is that ocular fixation was not controlled. Eye movements during the adaptation period might have reduced the amount of visual adaptation. Likewise, eye movements during the presentation of the probe stimuli might affect the aftereffect strength. However, there is no reason why eye movements should distort certain conditions selectively. In other words, the potential confound of eye movements can affect conditions in a similar manner. 

A previous study has demonstrated that size adaptation affects perceived numerosity [31]. When the size adapter is smaller than a cloud of dots, observers overestimated the number of dots and vice versa for a size adapter that was bigger than the dot cloud. Bi-directional object repulsion provides an intuitive explanation for this finding. An adapter smaller than the cloud repels dots from each other, leading to the impression of more dots as objects occupying an area that is more extended in the visual field and which appears more numerous [32]. Density judgments are not affected by size adaptation [31], suggesting that density estimates are derived from a neural representation that is independent of the adaptation effect, probably in extra-striae areas [33,34,35,36]. This is consistent with the findings in the present study, showing that density adaptation is not responsible for repulsive effects on space. 

All these experiments demonstrate that visual adaptation affects apparent space by inducing a repulsive aftereffect in localization. Spatial repulsion of objects through lines is sufficient to explain adaptive spatial distortions in visual adaptation. 

## Data Availability

Data used for this publication have been deposited into the Open Science Foundation repository: https://osf.io/9yevd/?view_only=d5d1040f63a64078a6e8ce6f968d2fa4, accessed on 16 April 2020.
